# Hydrodynamic schooling of flapping swimmers

**DOI:** 10.1038/ncomms9514

**Published:** 2015-10-06

**Authors:** Alexander D. Becker, Hassan Masoud, Joel W. Newbolt, Michael Shelley, Leif Ristroph

**Affiliations:** 1 Applied Math Lab, Courant Institute, New York University, 251 Mercer Street, New York, New York 10012, USA

## Abstract

Fish schools and bird flocks are fascinating examples of collective behaviours in which many individuals generate and interact with complex flows. Motivated by animal groups on the move, here we explore how the locomotion of many bodies emerges from their flow-mediated interactions. Through experiments and simulations of arrays of flapping wings that propel within a collective wake, we discover distinct modes characterized by the group swimming speed and the spatial phase shift between trajectories of neighbouring wings. For identical flapping motions, slow and fast modes coexist and correspond to constructive and destructive wing–wake interactions. Simulations show that swimming in a group can enhance speed and save power, and we capture the key phenomena in a mathematical model based on memory or the storage and recollection of information in the flow field. These results also show that fluid dynamic interactions alone are sufficient to generate coherent collective locomotion, and thus might suggest new ways to characterize the role of flows in animal groups.

## Abstract (web summary)

Schooling or flocking of animals in nature is generally explained via an energy optimization approach. Here, Becker *et al*. mimic the conditions for an infinite array of swimmers in a water tank and show that fluid dynamic interactions alone are sufficient to lead to coherent and collective locomotion.

## Graphical abstract

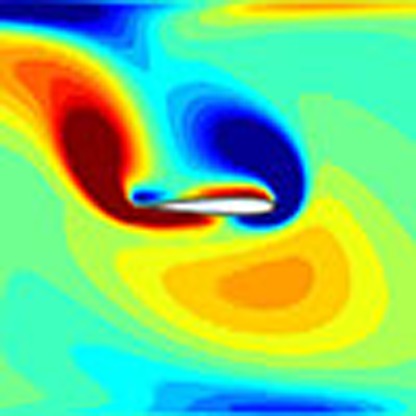

When collections of bodies move within a fluid, the motion of each is influenced by the flows generated by others, often with surprising and important consequences. During sedimentation of particulate suspensions, for example, many-body fluid-mediated interactions lead to unusual particle trajectories as well as large-scale flows that significantly affect the global settling time^1^. Similar interactions are at work in many natural contexts and engineering applications, from water droplets within clouds^2^ to beds of sand or other granular matter suspended by flows^3^. These cases in which bodies passively respond to external forcing are complemented by systems in which the constituents actively generate the flows through which they interact^4^,^5^,^6^. To date, studies of such active suspensions or active matter have largely focused on swimming micro-organisms, or micro-particles, that produce flows through chemical reactions^7^,^8^,^9^. At such small scales, viscosity dominates inertia, flow fields are established nearly instantaneously, and thus interactions between bodies can be thought of as immediate and depending only on the present configuration and motions^10^.

For larger bodies and faster motions where fluid inertia is important, flows decay slowly, and interactions can no longer be viewed as instantaneous. The biological realm provides some of the most fascinating examples in which individuals actively generate inertial flows, such as schooling of fish and flocking of birds. The collective behaviours are intriguing from many perspectives^11^,^12^,^13^,^14^, including the conventional fluid dynamic view that highly ordered animal groupings benefit from flow-mediated interactions by saving on the energetic cost of movement^15^,^16^. For example, V-formation flight of birds is thought to involve favourable interactions with the up- and down-wash of upstream neighbours^16^,^17^,^18^, and correlations in flapping motions and inter-neighbour spacing in actual flocks have been interpreted as behavioural responses that take advantage of these flows^19^,^20^. For fish schools, a diamond-shaped lattice arrangement is thought to enable individuals to extract energy from the vortices shed by others^15^,^21^, although field and laboratory studies have led to conflicting conclusions regarding a possible hydrodynamic function of schooling^22^,^23^.

Inspired by animal groupings, here we seek to understand how fluid-mediated interactions among self-propelling bodies influence their collective locomotion. Specifically, we study such interactions in the idealized setting of linear arrays of wings undergoing prescribed flapping motions but whose swimming speed is free or dynamically determined. Because the flows are inertial or long-lived, the forcing experienced by each body depends on the dynamical history of the ensemble, and the system can be said to possess memory of past states. We aim to understand how this effect is manifest in collective locomotion and to draw parallels to other fluid-structure systems that display memory^24^,^25^,^26^. Understanding these interactions could also provide insight into the fluid dynamics of animal groups on the move, especially with regard to possible exploitation of flows for formation locomotion or highly ordered groupings. Moreover, the underlying hydrodynamic principles might be put to use in applications involving the interactions of bodies with unsteady flows^27^,^28^,^29^,^30^,^31^,^32^. For example, schemes might be devised for harvesting energy from waves or other time-dependent flows, and air or water vehicles could be designed to take advantage of interactions between propulsors.

## Results

### Experimental approach

**Figure 1:**
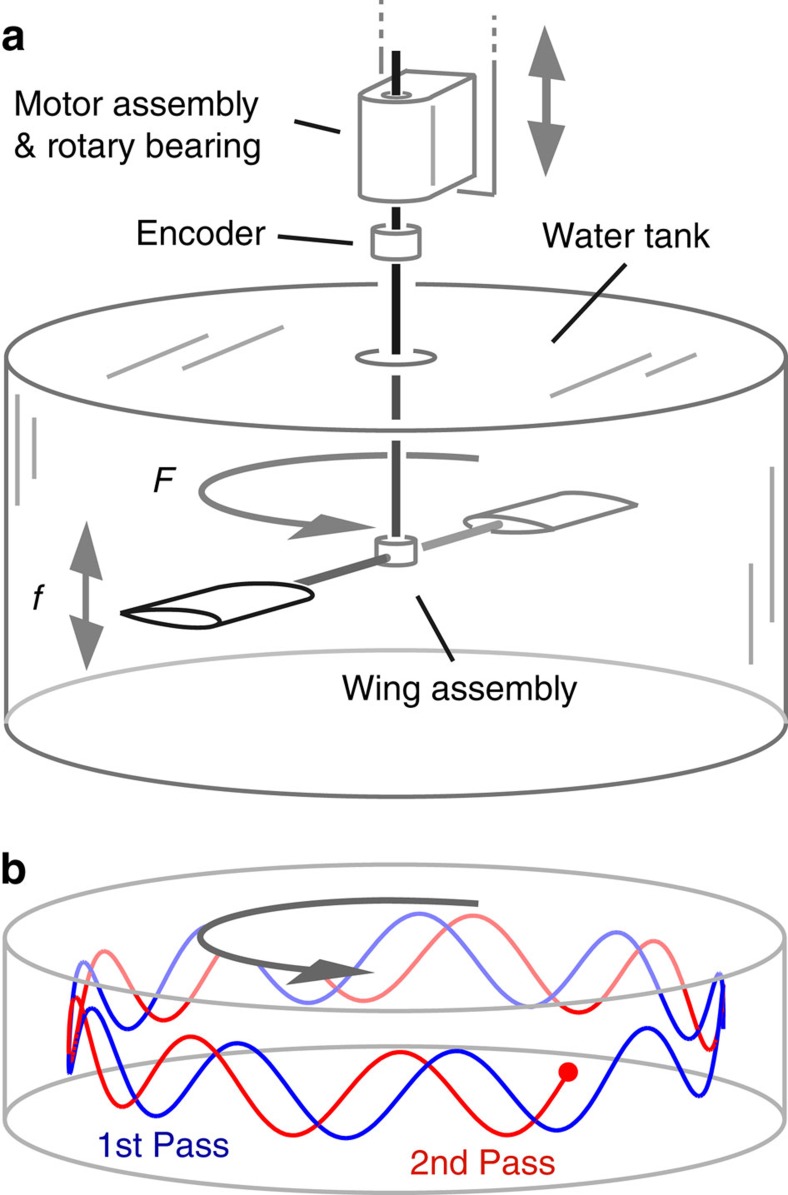
Flapping wings swimming in rotational orbits mimic an infinite array of locomotors. (**a**) A motor heaves a wing or wing pair up and down at prescribed frequency *f* and peak-to-peak amplitude *A*, resulting in swimming of rotational frequency *F* around a cylindrical water tank. (**b**) The rotational geometry allows for interactions with the flows generated in previous orbits.

Our experimental design is guided by the goals of achieving self-propulsion for prescribed flapping motions and realizing an effectively infinite array of bodies to explore their interactions. As shown in Fig. 1a and detailed in the Methods section, the device consists of one or more wings or hydrofoils that are heaved up and down, and consequently swim in orbits around a cylindrical water tank^33^,^34^,^35^. A video showing the device in operation is available in Supplementary Movie 1. Free swimming is achieved by mounting the wings to a vertical axle via a low-friction rotary bearing. Thus, the forward locomotion is not prescribed but is an outcome of the interaction with the fluid. This self-propulsion condition as well as the inertial or high Reynolds number flows are important properties shared with bird flight and fish swimming. Our system, however, involves fixed separations between bodies, whereas inter-individual spacing is free or dynamically determined in animal groups, and this difference will likely lead to differences in the emergent locomotion.

Previous studies have shown that the dynamics and flows observed in rotational systems compare well with those in translational geometries^33^,^34^,^35^,^36^. To ensure the phenomena we observe are not a peculiarity of geometry, we accompany our three-dimensional (3D) rotational experiments with computational simulations in two-dimensional (2D) translational domains, as discussed in detail below. Most importantly, the rotational geometry induces each wing to swim within the wakes of others, as indicated by the trajectories of Fig. 1b, and this arrangement thus mimics the conditions in an infinite array. The device also allows us to explore swimming behaviour for varying strength of hydrodynamic interactions between swimmers. Increasing the number of wings connected to the assembly decreases the spacing between members in an array and amplifies interactions. Further, increasing flapping frequency or amplitude induces faster swimming and thus stronger flows to be encountered in subsequent orbits. The swimming dynamics are characterized by measuring the rotational speed around the tank, and use of a clear-walled tank allows for visualization of the flow fields.

### Dynamics of interacting wings

**Figure 2:**
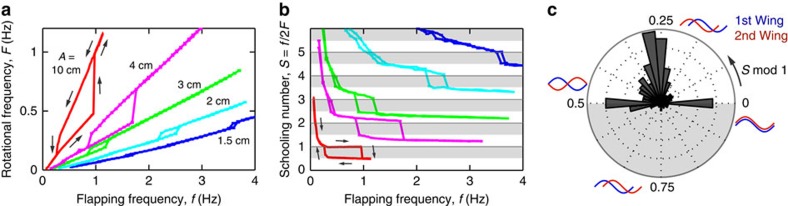
Dynamics of interacting wings. (**a**) Swimming speed, as measured by the rotational frequency *F*, versus flapping frequency *f*. For each peak-to-peak amplitude *A*, an upward sweep of *f* is followed by a downward sweep (as indicated by arrows), and the data form a hysteresis loop. (**b**) Schooling number *S*, which represents the number of wavelengths separating successive wings. Each hysteresis loop is bounded by in-phase (integer value of *S*) and out-of-phase states (half-integer *S*). (**c**) A polar histogram of *S* mod 1 shows peaks corresponding to preferred spatial phase relationships between successive wings.

Previous studies have used rotational systems to study the dynamics and hydrodynamics relevant to an isolated wing^33^,^34^,^35^. This is achieved by relatively slow swimming of widely spaced hydrofoils, in which case interactions are weak and the swimming speed follows simple dependencies on flapping frequency and amplitude. Here we aim to induce strong interactions by considering fast swimming of a wing pair, and indeed we observe markedly different dynamics. To systematically characterize the locomotion, we vary both the peak-to-peak amplitude *A* and flapping frequency *f* for the pair and measure the resulting rotational frequency *F* around the tank. For each *A*, we incrementally increase *f*, and this upward sweep is followed by a downward sweep to low values. The data of Fig. 2a show that faster flapping leads to faster swimming, although *F*(*f*) does not always change continuously. For *A*=10 cm, for example (red curve), *F* increases smoothly until a critical frequency near *f*=1 Hz, at which *F* abruptly increases, in this case doubling its speed. As *f* is increased further, *F* again increases continuously. For decreasing *f*, *F* remains high before abruptly dropping near *f*=0.2 Hz. Thus, the measured dynamics display a hysteresis loop, and different flapping amplitudes lead to other loops. This hysteretic behaviour is associated with bistability of states: for identical flapping kinematics, the array can take on one of two ‘gears' corresponding to slow and fast swimming modes.

To organize these observations, we first note that the quantity *f*/*F* represents the number of flapping strokes in one orbit of the tank, and thus the number of strokes separating the two wings is given by *S*=*f*/2*F*. Equivalently, this schooling number *S* represents the separation distance between successive swimmers measured in wavelengths of motion, and this quantity serves as a general way to characterize arrays of synchronized swimmers. Specifically, *S* encodes the spatial phase shift between successive swimmers: whole integer values of *S* denote spatially in-phase states in which neighbouring wings trace out the same path through space, and half-integer values indicate out-of-phase states. In Fig. 2b, we show how *S* depends on *f* for all the data of Fig. 2a. At each amplitude, low *f* leads to slow swimming or large *S*, which indicates weak interactions. Increasing *f* leads to decreasing *S* followed by saturation near a whole integer value, thus residing nearly in-phase for a broad range of frequencies. Further increasing *f* induces an abrupt downward jump in *S*. Conversely, when *f* is decreased, the system approaches half-integer *S* (out-of-phase) before an abrupt upward jump.

Viewing all amplitude data sets together reveals preferred and avoided ranges of *S* for strongly interacting wing arrays. We quantify this observation in Fig. 2c, in which the histogram of *S* mod 1 reveals the distribution of spatial phase shifts. Values between 0.5 and 1 are almost entirely absent, and among the prevalent phases, distinct peaks appear at values of 0, 0.25 and 0.5. Thus, these swimmers preferentially occupy spatial phase relationships corresponding to purely in-phase, leading by a quarter cycle, and purely out-of-phase. The appearance of these particular phase relationships suggests that schooling in this system involves bodies interacting coherently through their wave-like flow fields.

### Flow visualization

**Figure 3:**
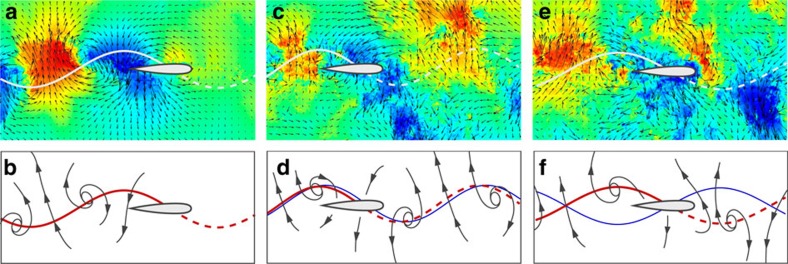
Flow visualization. (**a**,**b**) Flow field around a non-interacting wing of chord length 3 cm extracted using particle image velocimetry and rendered in a schematic. The colour map indicates the vertical component of the velocity vector field, with red indicating upward and blue downward flows. The upstroke produces an upward flow and the downstroke a downward flow. (**c**,**d**) Slow mode of interacting wings: the downstroke of a wing (red path) occurs within the downward flow of its predecessor (blue path). (**e**,**f**) Fast mode: the downstroke occurs within the upward flow of its predecessor.

To shed light on the hydrodynamics of these modes, we seed the fluid with particles and use high-speed video and image analysis to extract the flow fields (Supplementary Movies 2 and 3). First, we consider a single wing swimming at relatively low amplitude and frequency, in which case interactions are weak. Indeed, the flows produced are largely dissipated between successive passes, and thus the wing swims into quiescent fluid, as shown by the velocity vector field of Fig. 3a and the schematic of Fig. 3b. The wing leaves behind a stream-wise array of counter-rotating vortices that are staggered vertically. This so-called reverse von Karman wake has been observed in biological and physical experiments, and is a signature of thrust production during flapping^33^,^34^,^35^,^36^,^37^,^38^. The vortices are separated by strong up and down flows, which we highlight in Fig. 3a using a colour map of the vertical component of velocity. Each upstroke generates an upward flow (red) and each downstroke a downward flow (blue), and thus the wake can be viewed as a wave in space that reflects the trajectory of the wing.

To induce stronger interactions, we attach multiple wings to the assembly but maintain the same flapping motion. Under these conditions, the system exhibits a hysteresis loop associated with the slow and fast swimming modes. Figure 3c and d show the flow field for the slow mode, in which successive wings trace out similar paths through space. Captured here during the downstroke, a wing is seen to sink within the downward (blue) flow generated by its predecessors. Subsequently, the upstroke occurs within an upward (red) flow. This visualization reveals that not only are successive wings spatially in-phase with one another but also any given wing is also in-phase with the existing flow structure into which it swims. Thus, this constructive wing–wake interaction mode involves the repeated reinforcement of existing flows. Conversely, the fast mode involves antagonistic motions between each wing and the flow it encounters. As shown in Fig. 3e,f, the downstroke of the wing moves against the upward flow of its predecessor, and the subsequent upstroke occurs within a downward flow. This destructive mode thus involves the inversion of flow fields, in which the passing of a wing replaces an existing upward flow with a downward one, for example.

### Simulations

To gain additional insights into the intrinsic dynamics of swimmer arrays, we conduct computational fluid dynamics simulations that solve for the flow field and locomotion of a flapping body. Similar to the experiments, these simulations involve free swimming of a wing, where the emergent speed reflects hydrodynamic interactions (see Methods section as well as Supplementary Movies 4 and 5). The swimming dynamics is determined from the computed fluid forces, and an infinite array of swimmers is replicated by having a single wing repeatedly traverse a domain of length *L* with periodic boundary conditions^39^. Unlike the experiments, the flow field is 2D and the swimming motion is translational rather than rotational. Thus, observations common to both experiments and simulations are expected to be generic and not due to system dimensionality or geometry. We systematically explore the dynamics through a procedure similar to that of the experiments: Flapping frequency is incrementally increased, with each step initialized with the final data of the previous step and then allowed to reach a terminal swimming speed. This upward sweep of frequency is then followed by a downward sweep.

**Figure 4:**
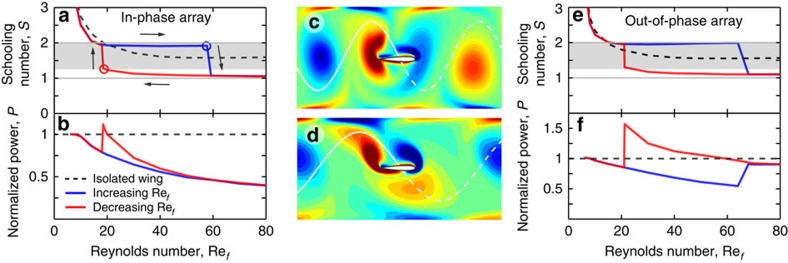
Simulations of interacting wings. (**a**–**d**) An infinite array of synchronized or temporally in-phase wings is simulated by a single airfoil driven to flap up and down, and allowed to swim freely left to right across a periodic domain. (**a**) Schooling number *S* for increasing (blue) and decreasing (red) flapping Reynolds number, Re_*f*_. A non-interacting wing (dashed curve) swims at a speed intermediate between the two schooling modes. (**b**) Input power normalized by that of an isolated wing. (**c**) Computed vorticity field for the slow mode (blue circle in **a**): The wing slaloms between vortices. (**d**) Fast mode (red circle in **a**): the wing intercepts each vortex core. (**e**,**f**) Schooling dynamics and power consumption for an array in which nearest neighbours flap temporally out-of-phase with one another.

These simulations reveal a number of features in common with the experiments, including the coexistence of different locomotion states for identical flapping motions. Figure 4a shows the complete characterization of the dynamics using *S*=*f*/*F*, where *F*=*U*/*L* is the traversal frequency. Here, the simulation results depend only on flapping Reynolds number Re_*f*_, which plays a role analogous to *f* in experiments (see Methods). Dynamical signatures of strong interactions again include bistability of states, a hysteresis loop, and avoided values of *S*, suggesting that these are general features of locomotor arrays. Similar to the experiments, these simulations display a downward jump at whole integer *S*, but unlike the experiments the upward jump seems to occur at a quarter rather than half integer, leading to a wider band of avoided phases. Nonetheless, the flow fields at these transition points reveal constructive and destructive characters, respectively. For the slow mode near Re_*f*_≈60 and *S*≈2, the vorticity plot of Fig. 4c shows that the wing slaloms between vortices, contributing vorticity of the same sign to each. For the fast mode near Re_*f*_≈20 and *S*≈1.25, Fig. 4d shows that the wing intercepts and destroys each oncoming vortex core. Interestingly, previous 2D experiments of flapping foils in unsteady flows have also shown vortex slaloming and interception modes^40^ (see Discussion section).

Importantly, these simulations allow us to compare the swimming speed and power input for interacting bodies versus an isolated, non-interacting wing. The latter case is simulated in a longer domain to ensure that terminal speed is reached in the absence of self-interactions (see Methods section). To compare speed, we plot as a dashed curve in Fig. 4a the computed *S* for an isolated wing, which is found to lie between the curves for the slow and fast modes. Thus, with respect to speed, hydrodynamic interactions can be beneficial or detrimental. To compare energetic cost, we use the computed vertical force and prescribed vertical velocity to form the average input power at steady state, and Fig. 4b shows the power investment in the presence of interactions normalized relative to that of a non-interacting wing at the same Re_*f*_. Surprisingly, both the slow and fast modes save power for nearly all Re_*f*_, and the presence of interactions can lead to savings of >50%. The slow mode always saves more than the fast, and the fast mode displays a moderate energetic increase relative to an isolated wing for a narrow range of Re_*f*_ near the upward transition point.

These simulations also allow us to explore how differences in temporal phases between swimmers in an array affect their dynamics and power consumed. We consider a linear array in which each wing flaps out-of-phase in time with its nearest neighbours (see Methods section for details). To compare these results to the previous case of a temporally in-phase array, we use a natural generalization of the schooling number based on the total phase shift due to both the temporal phase *ϕ*_T_ and spatial phase contributions: *S=ϕ*_T_/2*π*+*f*/*F*. Temporally in-phase arrays have *ϕ*_T_=0 and out-of-phase arrays have *ϕ*_T_=*π*, and for all cases this definition preserves the property that integer values of *S* represent spatially in-phase trajectories while half-integer *S* indicate spatially out-of-phase trajectories. In Fig. 4e, we characterize the locomotion of out-of-phase arrays using this modified schooling number, and we find that all the salient features of in-phase arrays are also present in this arrangement. Interestingly, important differences between in-phase and out-of-phase arrays arise in the power consumed, as seen by comparison of Fig. 4b,f. In particular, the fast mode for out-of-phase arrays is associated with higher power consumption, and indeed it is typically higher than that of an isolated swimmer. The origin for this difference is unclear but may be related to the more erratic and intense flows observed for the case of temporally out-of-phase arrays.

### Mathematical model

**Figure 5:**
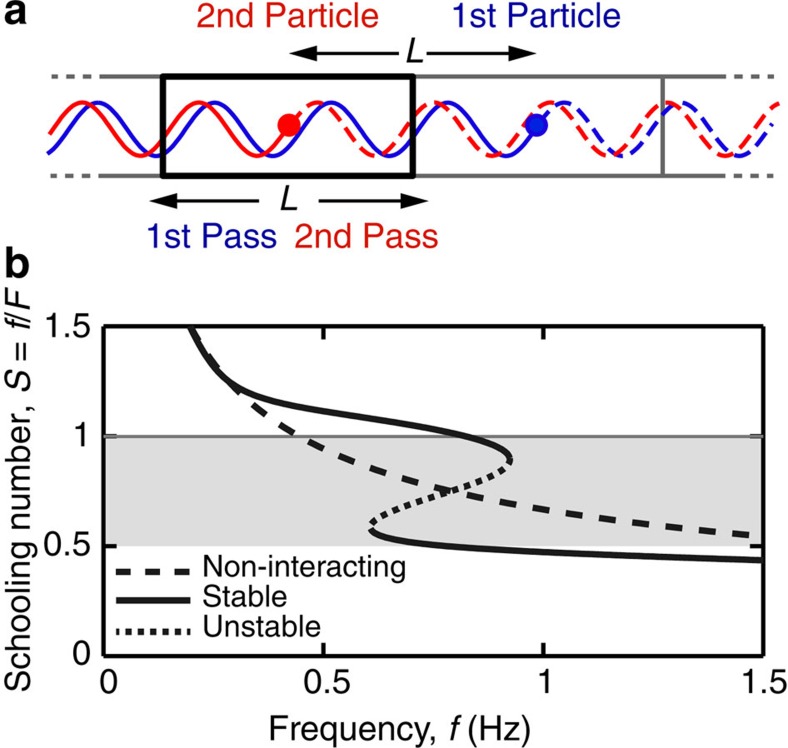
Mathematical model. (**a**) An infinite linear array of synchronized swimmers is represented by a single particle undergoing repeated passes across a domain specified by periodic boundary conditions. (**b**) Schooling number for a model with parameter values *s*=1.5, *P*=1.5, *ɛ*=1, *τ*=1 (see text for details).

Our experiments and simulations motivate a minimal model that describes the collective dynamics of a linear array of swimmers. As shown in Fig. 5a, an infinite array of bodies flapping in synchrony and spaced by a distance *L* is represented by a single body that repeatedly traverses a domain of length *L* that is specified by periodic boundary conditions. In our conception, the body's horizontal speed is perturbed because it encounters the wake produced in its previous pass through the domain. The perturbation strength depends on the traversal time *t*−*t*′, which is the time elapsed since the body was last at the same location: *X*(*t*)=*X*(*t*′)+*L*. Models of this type take the form of a delay differential equation for the swimming speed 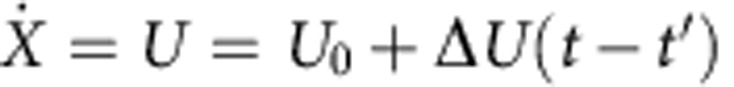. Here, *U*_0_ is the speed in the absence of interactions—that is, the speed of a single, isolated swimmer—and Δ*U* represents the perturbation due to wing–wake interactions. The effect of memory is explicitly incorporated through the time delay *t*′, which is not a constant but rather depends on the dynamical history.

Here we consider a specific model of this type given by the equation:


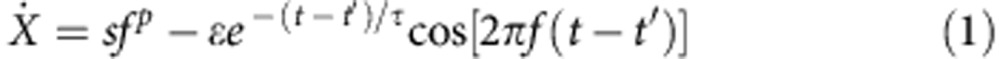


The first term describes how the speed of an isolated swimmer increases with flapping frequency *f*, where *s* and *p* are parameters. This power law dependence of speed on frequency is consistent with our measurements for a single wing. The second term represents the perturbation to the speed, where *ɛ* is the wing–wake interaction strength. Importantly, the perturbation depends on the difference 2*πf*(*t*−*t*′) in the current phase in the flapping cycle and the phase when last at the same location. One might expect that the forcing is a periodic function of this phase difference, and the cosine form, in particular, is found to yield model solutions that closely correspond to the experimental data (see below). Finally, the dissipation of flows, and thus weakening of interactions for longer traversal times, is captured by the exponential term with a decay timescale of *τ*.

We then seek steady swimming solutions corresponding to 
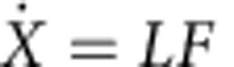
, where *F*=1/(*t*−*t*′) is the frequency with which the body crosses the domain. Putting these relationships in the above dynamical equation and taking *L*=1, we obtain a nonlinear algebraic equation relating *F* and *f*:


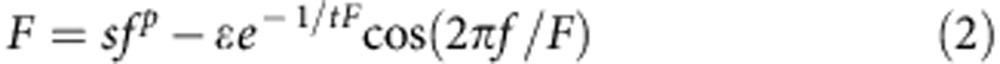


To illustrate the structure of the solutions, which are solved numerically, we display in Fig. 5b the schooling number *S*=*f*/*F* for a model with order-one parameter values, as given in the caption of Fig. 5. The solution curve *S*(*f*) displays a fold that consists of upper and lower stable branches (solid curves) connected by an unstable branch (dotted curve). The non-interacting swimmer (dashed curve) serves as a point of comparison.

At low *f*, the wing progresses slowly, *S* is large, and the solution resembles the non-interacting case. The fold appears at higher *f* and corresponds to the coexistence of a slow mode (*S*≈1) and a fast mode (*S*≈0.5), and at yet higher *f* the slow mode disappears. The speed of an isolated wing lies intermediate between these modes, as found in simulations. The model thus reproduces remarkably well the key observations from the experiments and simulations, suggesting that relatively simple interaction laws that include memory underlie the complex hydrodynamics of swimmer arrays.

## Discussion

Collectively, these findings indicate that the intrinsic dynamical states of locomotor arrays involve repeated and coherent interactions between each flapping body and the oscillating flow into which it swims. Physically, we interpret these modes as stable equilibria, that is, conditions for which thrust and drag balance to yield steady swimming and the system returns to its original speed if perturbed. Although the associated flow fields are spatially and temporally complex, experiments and simulations reveal a slow mode associated with constructive wing–wake interactions and a faster, destructive mode. These observations motivate a dynamical model that incorporates a forcing that depends on the relative phase between oscillations of the body and the oncoming flow, and the strong correspondence with experiments suggests that the fluid-mediated interaction laws have a tractable mathematical form.

The success and simplicity of our model also suggests that our findings are rather generic, and indeed previous studies of flapping bodies in unsteady flows show behaviour reminiscent of the interaction modes discussed here^28^,^30^,^40^,^41^,^42^,^43^,^44^,^45^. Of particular relevance are the experiments of Gopalkrishnan *et al*.^40^, which determine the conditions for which a flapping foil fixed within an oncoming flow interacts constructively or destructively with the unsteady drag wake of an upstream cylinder. Our experiments show that analogous modes exist for self-propelled bodies, which interact through thrust wakes and whose speed is not prescribed but is dynamically selected. Additional novel aspects of our work include the identification of such modes as stable equilibria of locomotion and the discovery of coexistence of modes for identical driving conditions.

Many topics remain for future studies, including an explanation of the differences between our 3D experiments and 2D simulations in the fine structure of the hysteretic dynamics. Further, while our simulations show that temporal variations in swimming speed are typically <10%, future studies involving bodies of lower density relative to the fluid will assess how stronger fluctuations affect the dynamics. It will also be of interest to observe the modes assumed by arrays in which the inter-wing spacing is not fixed but is dynamically selected. We also aim to better understand how the power consumed is linked to the locomotion modes, a topic not addressed by the experimental measurements and mathematical modelling presented here. Ultimately, a long-term goal of this line of research is a derivation of schooling interaction laws directly from the relevant hydrodynamics.

Because the intrinsic modes of interaction represent collective locomotion in the absence of active behavioural response, they provide a purely physical system to which highly ordered animal groups can be compared. Previous studies of schools have not included all of the measurements necessary to determine spatial phase; however, when swimming in the wake of a fixed cylinder, fish adopt flapping motions that are spatially in-phase with the oscillating flow^46^,^47^. In the language introduced here, this behaviour corresponds to integer values of the schooling number, *S* mod 1=0. For formation flight of birds, recent measurements show that individuals are either out-of-phase (*S* mod 1=0.5) or one quarter phase (*S* mod 1=0.25) with respect to the flow produced by an upstream neighbour^20^. In light of our results, we interpret these particular locomotor-flow phase relationships as suggesting that coherent fluid-mediated interactions play a role in determining group structure. For formation flight or swimming of animals, perhaps the emergent structure represents a stable configuration in which not only speed but also inter-individual spacing are dynamically determined^45^. Future work might explore this idea by measuring the temporal flapping phase, spacing and swimming speed—and thus schooling number *S—*in laboratory realizations of fish schools^48^. A narrow distribution of *S* across individuals would serve as evidence for coherent fluid-mediated interactions.

It is also interesting to speculate how the coexistence of, and transitions between, locomotion states might impact animal collectives. Proximity to a transition point would facilitate abrupt changes in the dynamics of the entire group^49^, which could play a role in the rapid and seemingly coordinated response of a school to a predator, for example. On the other hand, aggregates may be susceptible to ‘phase separation' or division into slow and fast subgroups, leading to a loss of group cohesion due to fracture or collisions.

Viewing schooling as the interaction of locomoting bodies through a fluid, our results highlight the critical importance of memory or history dependence. That is, the past state of the system is encoded in the flow field, and the present state is driven according to this stored information. This effect is explicitly included in our model as the time-delayed interaction term and manifested in the hysteretic dynamics. In contrast, history dependence is not relevant to the swarming of micro-organisms and active micro-particles, where the low Reynolds number and absence of inertia ensure effectively instantaneous interactions^4^,^5^,^6^,^7^. This effect is also not included in current statistical mechanical models of animal collectives, which instead specify interaction rules that depend only on the current state and act instantaneously^50^,^51^,^52^,^53^. Incorporating memory into such models could allow the field of active matter to be extended to systems in which inertia is important. We also note that memory effects can be important even for the case of a single locomoting body^33^,^36^,^43^,^54^,^55^. For flapping flight, for example, this might take the form of interactions between a wing with the vortices generated during previous strokes^54^.

Finally, we note that some elements of the schooling problem considered here may be shared with other systems that involve the close interaction of a body with a wave it produces. For example, intriguing parallels can be drawn to a recently studied model system that consists of fluid droplets bouncing on a vertically vibrating bath^24^. Vibration maintains an air layer between the drop and surface, and prevents coalescence, and the bouncing motion induces waves on the bath through which droplets can influence one another. Interaction effects include orbital motions of a pair, and memory is manifested even by a single droplet that, under appropriate conditions, can coherently interact with its own self-generated wave to induce ‘walking' or horizontal motion^24^,^25^,^26^. The dynamical description of this problem shares salient features with our mathematical model, including memory-dependent forcing and coherent body–wave interactions^26^. In-phase schooling states in our rotational experiments are also reminiscent of the Bohr model of the atom, which associates electron orbits around the nucleus with standing wave modes. Indeed, as in the pilot-wave interpretation of quantum mechanical systems^56^, a self-interacting swimmer can be viewed as having particle (wing) and wave (wake) identities whose mutual influence leads to coherent states of motion.

## Methods

### Experiments

Experiments use 3D-printed plastic airfoils (NACA0017) of chord length *c*=6 cm and span 15 cm. Wings are centred at a distance of 26 cm from the driving axle, and the tank measures 46 cm in radius and height. Similar to bird flight and fish swimming, the wings experience high flapping Reynolds numbers, Re_*f*_=*ρfAc/μ∼*10^2^−10^4^, and typical parameters include frequency *f∼*0.1−5 Hz and peak-to-peak amplitude *A∼*1−10 cm. Water density and viscosity are *ρ*=1 g cm^−3^ and *μ*=10^−2^ g cm^−1^ s^-1^. Rotational speed around the tank is measured using an optoelectronic encoder. Flow visualization is carried out in a smaller system: wings of chord *c*=3 cm are held at a distance 8.5 cm in a clear tank of radius and height 15 cm. To visualize the flow fields, we seed the water with micro-particles, illuminate a cross-section of the tank with a laser sheet, capture high-speed video of the particle motion and apply a particle image velocimetry algorithm^57^.

### Simulations

The simulations use a Fourier spectral method with volume penalization^58^,^59^ to solve the 2D Navier–Stokes equations for a fluid of density *ρ* and viscosity *μ*. A NACA0010 airfoil of chord *c* and density 10 *ρ* is given a prescribed vertical motion of (*A*/2) cos 2*πft*, with peak-to-peak amplitude *A*=2*c*. The integrated fluid forces and Newton's second law determine the horizontal dynamics of the wing. The horizontal dimension has periodic boundary conditions, and the vertical dimension has height 3.5*c* and is bounded by walls. With these specifications, the results depend only on the flapping Reynolds number Re_*f*_=*ρfAc/μ∼*10−10^2^. The flow equations are solved in the frame of the wing on a 256 × 256 grid with time step 2 × 10^−6^. Simulations of a temporally in-phase array involve a single wing traversing a domain of length *L*=6*c*. The temporally out-of-phase case is carried out by simulating a wing pair each in a domain of length *L*=4*c*, with periodic boundary conditions being applied over the total length of 8*c*. Swimming of an isolated foil is simulated using a domain of length 24*c* with uniform velocity enforced at the inlet and outlet. The schooling number for an isolated wing (dashed curves in Fig. 4a,e) is defined to be *S*=*ϕ*_*T*_/2*π*+*fL*/*U*, where *ϕ*_T_ is the temporal phase (0 for in-phase, *π* for out-of-phase), *U* is the terminal speed and *L* is the inter-wing spacing or domain length used in each case.

## Additional information

**How to cite this article:** Becker, A. D. *et al*. Hydrodynamic schooling of flapping swimmers. *Nat. Commun.* 6:8514 doi: 10.1038/ncomms9514 (2015).

## Supplementary Material

Supplementary Movie 1 Operation of the experimental device in which wings are flapped up and down and free to swim around a cylindrical water tank.

Supplementary Movie 2 Flow visualization using microparticles illuminated by a laser sheet for the case of a single, non-interacting wing.

Supplementary Movie 3 Flow visualization for wings interacting in the fast mode.

Supplementary Movie 4 Simulations of a wing swimming in a periodic domain, here in a slow mode in which it slaloms between vortex cores

Supplementary Movie 5 Simulations of the fast mode in which the wing intercepts vortex cores.
